# Therapeutic Drug Monitoring of Antiseizure Medications Using Volumetric Absorptive Microsampling: Where Are We?

**DOI:** 10.3390/ph14070627

**Published:** 2021-06-29

**Authors:** Annachiara D’Urso, Marcello Locatelli, Angela Tartaglia, Linda Molteni, Cristian D’Ovidio, Fabio Savini, James Rudge, Ugo de Grazia

**Affiliations:** 1 Laboratory of Neurological Biochemistry and Neuropharmacology, Fondazione IRCCS Istituto Neurologico Carlo Besta, 20133 Milano, Italy; annachiara.durso@istituto-besta.it (A.D.); linda.molteni@istituto-besta.it (L.M.); 2 Department of Pharmacy, University “G. d’Annunzio” of Chieti-Pescara, 66100 Chieti, Italy; m.locatelli@unich.it (M.L.); angela.tartaglia@unich.it (A.T.); 3 Section of Legal Medicine, Center for Advanced Studies and Technology (CAST), Department of Medicine and Aging Sciences, University “G. d’Annunzio” of Chieti-Pescara, 66100 Chieti, Italy; cristian.dovidio@unich.it; 4 Pharmatoxicology Laboratory, Hospital “Santo Spirito”, Via Fonte Romana 8, 65124 Pescara, Italy; fabio.savini@ausl.pe.it; 5 Neoteryx LLC, Torrance, 90501 CA, USA; jamesr@neoteryx.com

**Keywords:** volumetric absorptive microsampling, therapeutic drug monitoring, bioanalysis, whole blood analysis, antiseizure medications, blood-to-plasma ratio

## Abstract

Therapeutic drug monitoring (TDM) of antiseizure medications (ASMs) represents a valuable tool to establish an appropriate patient therapy, to collect important information about drugs’ interactions and to evaluate patient’s metabolic capabilities. In recent years, a new volumetric absorptive microsampling technique using VAMS^®^ technology and Mitra^®^ devices, consisting of a sampling technique for the collection of fixed-volume capillary blood, was developed. These new devices provide a new home-sampling technique for whole blood that has been spread out to simplify sample collection from finger-pricks. This review is aimed to compare published articles concerning the application of VAMS^®^ in epilepsy and to identify the strengths and improvement points for the TDM of antiseizure medications. VAMS^®^ allowed a minimally invasive blood sampling even in the absence of trained personnel. Good stability data have indicated that storage and delivery can be facilitated only for specific ASMs. Trueness and precision parameters have been evaluated, and the hematocrit (HCT) effect was minimized.

## 1. Introduction

Epilepsy is a chronic brain disease that affects approximately 50 million people worldwide. Etiology is very variable encompassing genetic forms, metabolic diseases, brain injuries etc. although a higher percentage of the disease remains with unknown causing agents. Epilepsy is characterized either by recurrent seizures or absences that arise from uncontrolled electrical discharges in different brain districts. Seizures involving the whole body are known as generalized seizures while partial seizure refers to episodes that affect only a single part of the patient’s body. Sometimes a patient may experience a short loss of consciousness [1,2].

Seizures can be controlled, and the main treatments are antiseizure medications (ASMs). Antiseizure medications have complex pharmacokinetics (PK) and pharmacodynamics (PD), and they have shown high interindividual variations in dose–response relationships. Moreover, for some ASMs (carbamazepine (CBZ), phenobarbital (PB), phenytoin (PHT), and valproic acid (VPA)) narrow therapeutic indices were observed [3]. Cytochrome P450 enzymes family metabolizes most of the ASMs in the liver and this may result in metabolic interactions among different ASMs and/or other concomitant drugs [4]. In order to assess these interactions and monitor ASM efficacy and toxicity, TDM is usually performed in patients with epilepsy. TDM is also useful to monitor patient compliance with a therapy that is often life-long.

TDM may have a positive effect on the treatment, especially in vulnerable groups such as children, the elderly, and pregnant women [5,6]. Currently, TDM is a necessary tool for individualizing drug treatment and optimizing patient outcomes. In fact, by means of TDM, based on the patient’s outcome and the pharmacokinetic/pharmacodynamic properties of a drug, it is possible to optimize therapy in terms of both the effectiveness and the toxicity of the drug itself. Furthermore, TDM makes it possible to highlight both inter-individual variations in the dose–response relationship to a certain ASM as well as possible pharmacological interactions between different ASMs and/or other concomitant drugs. Many first-, second-, and third-generation ASMs display such interactions [4,7,8]. Only 70% of patients with epilepsy respond to treatment with one or two ASMs [9]. The remaining 30% require a more complex therapy based on the use of more than two ASMs and different drug combinations over time to achieve seizure control. Obviously, this polytreatment strategy can easily induce pharmacokinetic interactions, some of which are well known [3,4,10]. Moreover, the pharmacokinetic properties of ASMs are influenced by a large number of factors such as age-related decrease in the ability to metabolize the drug, individual genomic variability in metabolism, gender, lifestyle and dietary habits, and comorbidities [4,7,10]. Therefore, and since dose–response relationships may change over time, TDM is the necessary tool to make ASM treatment highly patient-specific, even in the case of multi-drug treatments or polypharmacy.

The most-used matrices for drug concentration quantification are blood, plasma, serum, cerebrospinal fluid (CSF), urine, and saliva. Among these, plasma and serum are normally used for ASMs concentration determination. These matrices have been obtained from venous blood (sample volume ranging from 500 µL to 5 mL); thus, blood sampling requires a collection by trained personnel for venipuncture procedure. In addition, venipuncture presents other drawbacks: (i) it is invasive sampling, (ii) it requires specific storage conditions, and (iii) large volumes of samples are required. All of these factors have led to the development of alternative sampling techniques that overcome the above-listed drawbacks and require less sample volume (<50 µL). The use of microsampling, sample pretreatment strategies, and the fact that high sensitivity modern instrumentation (LC–MS and LC–MS/MS) can be highly automated, makes this a practical strategy. Regarding microsampling techniques, dried blood spot (DBS) is the most common. This sampling approach is feasible, low-cost, and well documented, having been in use for over 50 years. In 1963, Guthrie and Susi [11] were the first to demonstrate the usefulness of a microsampling technique. They used dried blood samples to detect phenylketonuria in a population of newborn infants by measuring phenylalanine concentrations in whole blood. This technique has been included as routine technique for the screening of newborn infants for diagnosis of metabolite disorders.

**Figure 1:**
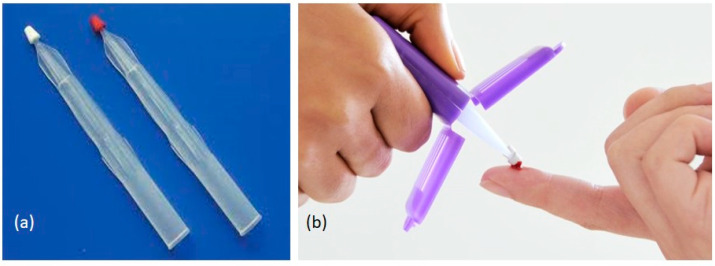
Panel (**a**) on the left, a Mitra^®^ device before whole blood loading, on right a device already loaded with blood; panel (**b**) loading capillary blood on Mitra^®^ device.

Although DBS works well especially for semiquantitative measurements, there are two challenges with DBS. The first is the quality of the sample where it has been reported that insufficient spot quality led to poor results. The second reported challenge with DBS revolves around the hematocrit (HCT). The greater the percentage HCT the less the blood spreads on the DBS paper which then leads to positive biases in the data. A new and simple approach to blood collection that includes all the advantages of DBS sampling but overcomes the aforementioned issues is represented by volumetric absorptive microsampling (brand name Mitra^®^ devices and VAMS^®^ technology; Neoteryx, Torrance, CA) [12,13]. Mitra^®^ devices with VAMS^®^ technology (Figure 1) consist of a plastic sampler, to which a polymeric, absorptive tip is connected, allowing the easy collection of a fixed volume of blood (10 µL, 20 µL, or 30 µL) after finger prick by a lancet. The approach involves the absorption of a liquid sample onto a porous substrate by wicking, through capillary forces, where the volume of sample absorbed is controlled by the properties and amount of substrate. Compared to traditional collection, VAMS^®^ requires a small blood volume (from 10 µL to 30 µL), a less invasive procedure, reducing the risk of infection. Since the withdrawal can be made in the absence of qualified personnel, it allows for sample collection at home. Today, VAMS^®^ may represent a very promising microsampling technique (reviewed in [14,15,16]) (a complete list of published papers involving VAMS^®^ use can be found in [17]). As discussed in ref [12] and [13] VAMS^®^ are reliable tools for pharmacokinetic and toxicokinetic studies. Protti et al. [18] have recently published a very useful tutorial on VAMS^®^ use. In this paper are discussed all stages of VAMS^®^ procedures comprising the use starting from different matrices. Authors also discussed VAMS^®^ applications and the possibility to automate VAMS^®^ analysis in the near future. Very recently, a paper from Harahap and colleagues [19] discusses the advantages and challenges that might be found in the use of VAMS^®^ as an alternative sampling tool in clinical trials and TDM during the COVID-19 pandemic.

The purpose of this review was to compare the recently reported VAMS^®^ methods for quantifying a number of antiseizure medications including cannabis derivative cannabidiol (CBD), focusing on sample preparation, stability, HCT effect, and plasma values correlation. The PubMed database has been checked with the following research entry: [therapeutic drug monitoring] AND [antiepileptic drugs] OR [cannabidiol] AND [Volumetric Absorptive Microsampling] OR [capillary microsampling]. In the search strategy, the term [antiepileptic drugs] was preferred to ASMs because the last is not very common so far, although we believe it is more appropriate according with the paper from French JA et al. [20]. The antiseizure medications considered in this review are those illustrated in Table 1 and namely: brivaracetam (BRV), carbamazepine (CBZ), and its pharmacologically active metabolite carbamazepine-epoxide (CBZ-E), ethosuximide (ETS), felbamate (FBM), gabapentin (GBP), lacosamide (LCM), lamotrigine (LTG), levetiracetam (LEV), oxcarbazepine (OXC), and its pharmacologically active metabolite 10-OH-monohydroxycarbazepine (10-OHOXC), perampanel (PMP), phenytoin (PHT), phenobarbital (PB), pregabalin (PGB), primidone (PRM), rufinamide (RFN), topiramate (TPM), valproic acid (VPA), and zonisamide (ZNS).

Cannabidiol was approved by the European Medicines Agency as additional treatment for Dravet syndrome and Lennox-Gastaut syndrome in July 2019 [21,22]. Usually, these severe forms of childhood refractory epilepsy are treated with clobazam. The introduction of CBD in managing these syndromes is, in our opinion, a new important step in the use of cannabis derivatives to treat epileptic syndromes and other diseases. Following this approval, CBD was also included in this review because (i) its emerging role as ASM [22] and (ii) the possibility of use of VAMS^®^ in TDM of this drug [23,24,25].

Articles have been filtered for publication in the last five years. This search strategy retrieved six eligible publications.

The applied eligibility criteria were: validation of a new bioanalytical methods for ASMs drug monitoring by mean of Volumetric Absorptive Microsampling (based on Mitra^®^ devices with VAMS^®^ technology), applied only to human patients.

## 2. Volumetric Absorptive Microsampling Analysis Using VAMS^®^ Technology

Volumetric absorptive microsampling involves the absorption of a liquid sample onto a porous substrate by transpiration (illustrated in Figure 1). The Mitra^®^ device with VAMS^®^ technology is designed to be simple and ergonomic to use. Sample preparation took place always after a drying period of around 1–3 h. The procedure involves different steps. The sample preparation, the stability of the device and the HCT effect have been optimized and studied, and some examples were reported in the following paragraphs. VAMS^®^ sampling in feasibility studies, such as those illustrated in this review, in most cases is performed by contacting the blood surface (avoiding full immersion), for a few seconds with the VAMS^®^ tip, using a standard venous blood tube. To date, studies involving actual sampling from the finger are still rare. Among those analyzed in this review, only two studies make use of finger pricking or capillary blood for ASMs [24,26]. In 2019, Sciberras et al. [27] published a pharmacokinetic study of radiprodil oral suspension in healthy adults comparing conventional venous blood sampling with two microsampling techniques, comprising VAMS^®^ technology. Radiprodil (UCB3491) is a new ASM that is currently under development. This drug acts on NMDA receptors as negative allosteric modulator. So far it has been used for treatment of infantile spasms (IS), a severe infantile seizure disorder, in which onset of seizures usually occurs within the first year of life. PK profile of radiprodil was analyzed either with conventional plasma measurement or VAMS^®^ technique with comparable results. The authors stated that the possibility of using VAMS^®^ technology microsampling would support future radiprodil pediatrics studies.

### 2.1. Sample Preparation

Sample preparation usually takes place after a drying period of the Mitra^®^ device (often at room temperature) ranging from 1 h to 3 h, sometimes in the presence of a bag with desiccant. Velghe and co-workers [26] have described VAMS^®^ and DBS microsampling for therapeutic drug monitoring of antiseizure medications in children with Nodding syndrome and epilepsy. Nodding syndrome is a highly debilitating generalized seizure disorder, usually affecting children between the ages of 5 and 15 in sub-regions of sub-Saharan Africa. The pharmaceutical treatment in patients with Nodding syndrome mainly include the first-generation antiseizure medications such as VPA, CBZ, PHT, and PB. The VAMS^®^ procedure reported by Velghe et al. [26] involved the sample collection in the morning by mean of finger pricking, immediately before the first medication of the day. The samples once dried (approximately 2 h) were stored at −20 °C. The Mitra^®^ samples were then extracted in 100 µL of acetonitrile/water (80:20, *v*/*v*) mixture, containing 5 mM ammonium acetate and deuterated internal standard. After shaking for 10 min at 60 °C and centrifuged, supernatant was diluted with 5 mM ammonium acetate (1:1, *v*/*v*), before the LC–MS/MS analysis. Pigliasco et al. [24] adopted a similar strategy in order to monitor CBD in children affected by Lennox-Gastaut syndrome and Dravet syndrome. To ensure proper sampling, patient’s finger was disinfected prior pricking with a microneedle and then the first drop of mixed blood and interstitial fluid was removed. Only the second drop of blood was collected after placing in contact with the VAMS^®^ tip of the Mitra^®^ device. This strategy avoids diluting whole blood with interstitial fluids and must be considered very carefully when planning TDM studies based on VAMS^®^ sampling.

Canisius et al. [28] have reported a clinical verification of volumetric absorptive microsampling for therapeutic drug monitoring of anti-epileptic drugs. In this study, different ASMs (10-OHOXC, CBZ-E, CBZ, OXC, PRM, PHT, ETS, PB, GBP, TPM, VPA, LTG, PGB, LCM, LEV, and BRV) were determined in whole blood collected by VAMS^®^. Samples were prepared by dipping the Mitra^®^ microsampler devices into the ASM-spiked whole blood. After sampling, the devices were dried for 2 h at room temperature (RT), and stored at RT unless specified otherwise. Extraction was performed adding a mixture of 54 µL of MilliQ-water with 40 µL of methanol working solution. After 45 min of shaker and centrifuge, 25 µL of supernatant was diluted into 350 µL of water (for samples containing VPA, ETS, and PB 50 µL were diluted into 75 µL of water), before the LC–MS/MS analysis. Velghe et al. in their first study [29] adopted the same strategy on ASMs-spiked whole blood.

D’Urso and colleagues [30] also have reported a new method to quantify 14 different antiseizure medications (LEV, LCM, ETS, RFN, ZNS, FBM, LTG, OXC, CBZ, PB, PRM, PHT, TPM, and PMP) and 2 active metabolites (10-OHOXC and CBZ-E) in samples collected by volumetric absorptive microsampling. Samples were collected by dipping only the lower part of the VAMS^®^ tip of Mitra^®^ device, in order to avoid oversampling, into whole blood in K3-EDTA containing tubes. Few seconds after the tip’s surface became entirely red, time necessary to ensure complete blood absorption; tips were removed and desiccated at least one hour at room temperature. Subsequently, dried tips were rehydrated in a 96-well plate with 200 μL of LC-MS/MS grade water and 250 µL of acetonitrile containing deuterated internal standards were added. Samples were then extensively mixed in an orbital shaker for 30 min and then centrifuged in 1.5-mL polypropylene conical tube. Supernatants diluted with mobile phase were vortex mixed in a glass vial and then injected into the LC–MS/MS system.

Velghe et al. [29] and D’Urso et al. [30] both highlight the need to avoid overfilling of the Mitra^®^ devices not completely immersing the VAMS^®^ tip into the blood.

The sample preparation strategy described by Moorthy et al. [23] in their study involves drying the sample for 60 h at room temperature, in a special dryer with desiccant gel, covered with aluminum foil. Then samples are extracted with 250 µL of a mixture consisting of 0.1 M zinc sulfate, 0.1 M ammonium acetate and 0.1% formic acid in acetonitrile. Samples were then vortex mixed, sonicated and finally centrifuged (4000 rpm) at 4 °C for 15 min. The entire diluted supernatants (500 μL) were then transferred to a solid phase extraction (SPE) plate for sample cleanup before injection Supernatants diluted (1:1 (*v*/*v*)) with water or mobile phase were injected in LC–MS/MS system.

In the study from Pigliasco et al. [24] VAMS^®^ samples were extracted with 200 µL methanol, sonicated and centrifuged. Extracted samples were then purified with an SPE online purification system.

The common features among the reported studies are the intensive laboratory trial to set different screening assays to optimize the extraction conditions from VAMS^®^ and the subsequent trial to set an appropriate analytical method. Often two or more organic solvents (i.e., methanol and acetonitrile) were tested in different combinations combined with different steps of re-hydration and/or sonication.

All the validated and reported procedures involve the use of water and an organic solution as precipitating agent, and a shaking step that varies from 10 min to 45 min or a sonication treatment. In addition, very different extraction temperatures were investigated (data illustrated in Table 1).

Common steps in sample preparation are: sample drying for an appropriate length of time (at least 2 h), extraction of drugs from tips polymer by the mean of rehydration combined with protein precipitation. Of particular significance, at least in our hands, was the rehydration step to ensure the minimum possible variability in extraction recovery. No further purification steps were needed, apart from a dilution in water or mobile phase in order to achieve a good and reproducible signal. Only studies on CBD monitoring, reported further purification steps, namely SPE plates [23] or SPE online [24].

All methods showed that the extraction recovery percentage values met European Medicines Agency (EMA) guidelines [31] for method development.

**Table 1:** Summary of investigated ASMs by mean of VAMS^®^ technology, extraction methods and blood/plasma ratio.

Analyte	VAMS^®^	Extraction Method	Extraction Temperature	Blood/Plasma Ratio (R)	Ref.
	Volume				
**10-OH-oxcarbazepine (10-OHOXC)**	10 µL	PP	RT	1.1	[30]
			RT	Nd	[28]
**Brivaracetam (BRV)**	10 µL	PP	RT	Nd	[28]
**Cannabidiol (CBD)**	20 µL	SPE	RT	0.696–0.827	[23]
	30 µL	PP + online purification	RT	Nd	[24]
**Carbamazepine (CBZ)**	10 µL	PP	22 °C, 60 °C	1	[29]
			60 °C	1.21	[26]
			RT	1	[30]
			RT	Nd	[28]
**Carbamazepine-epoxide (CBZ-E)**	10 µL	PP	22 °C, 60 °C	Nd	[29]
			RT	1.3	[30]
			RT	Nd	[28]
**Dihydroxy carbazepine**	10 µL	PP	RT	Nd	[28]
**Ethosuximide (ETS)**	10 µL	PP	RT	1.1	[30]
			RT	Nd	[28]
**Felbamate (FBM)**	10 µL	PP	RT	Nd	[30]
**Gabapentin (GBP)**	10 µL	PP	RT	Nd	[28]
**Lacosamide (LCM)**	10 µL	PP	RT	1	[30]
			RT	Nd	[28]
**Levetiracetam (LEV)**	10 µL	PP	RT	0.9	[30]
			RT	Nd	[28]
**Lamotrigine (LTG)**	10 µL	PP	RT	1.4	[30]
			RT	Nd	[28]
**Oxcarbazepine (OXC)**	10 µL	PP	RT	1.1	[30]
			RT	Nd	[28]
**Phenobarbital (PB)**	10 µL	PP	22 °C, 60 °C	0.9	[29]
			60 °C	0.93	[26]
			RT	1	[30]
			RT	Nd	[28]
**Phenytoin (PHT)**	10 µL	PP	22 °C, 60 °C	0.7	[29]
			RT	1.1	[30]
			RT	Nd	[28]
**Perampanel (PMP)**	10 µL	PP	RT	0.6	[30]
**Pregabalin (PGB)**	10 µL	PP	RT	Nd	[28]
**Primidone (PRM)**	10 µL	PP	RT	1.1	[30]
			RT	Nd	[28]
**Rufinamide (RFN)**	10 µL	PP	RT	1.5	[30]
**Topiramate (TPM)**	10 µL	PP	RT	1.5	[30]
			RT	Nd	[28]
**Valproic acid (VPA)**	10 µL	PP	60 °C	0.66	[26]
			RT	Nd	[28]
**Zonisamide (ZNS)**	10 µL	PP	RT	2.7	[30]
			22 °C, 60 °C	0.7	[29]

PP: protein precipitation; SPE: solid phase extraction; RT: room temperature; Nd: not determined.

### 2.2. Analytical Methods

#### 2.2.1. Traditional ASMs

The four examined studies [26,28,29,30] all rely on ASMs detection by mean of triple quadrupole LC-MS/MS analysis with similar mobile phases and reversed phase C18 columns (see Table 2). Drugs were detected with electrospray ionization (ESI) or heated-electrospray ionization (H-ESI) ionization probe, either in positive or negative mode with comparable accuracy, precision, and recovery. Clinical method validation was carried out comparing data obtained from Mitra^®^ devices equipped with VAMS^®^ tips with routine methods on plasma/serum routinely used in respective laboratories. Canisius et al. [28] and D’Urso et al. [30] compared results with already existing LC–MS/MS validated methods while Velghe et al. [29] compared their results with serum concentrations obtained using chemiluminescent magnetic microparticle immunoassay technology (CMIA, Abbott Diagnostics). In every study, the bias from routine in-lab methods and VAMS^®^ samples was acceptable except for drugs with a blood/plasma partitioning ratios less or greater than 1. For more details, please see Section 2.5 entitled “Blood-to-plasma ratio”.

#### 2.2.2. Cannabidiol (CBD)

The two reported studies [23,24] both rely on the use of a triple quadrupole LC–MS/MS instrumentation. The two studies differ in the use of ionization probes. For example, Moorthy et al. [23] used an instrument equipped with an ESI probe, whereas, Pigliasco et al. [24] preferred to use an Atmospheric Pressure Chemical Ionization (APCI) probe. Results are comparable and methods were both validated although only Pigliasco et al. referred to international guidelines on bioanalytical method validation [31,32]. Data comparison was conducted against venipuncture-derived plasma values, obtained with a method developed previously by Pigliasco et al. [24] with addition of an on-line SPE purification. Five patients were included in the clinical validation step.

Moorthy et al. [23] developed a different strategy. Whole blood samples were spiked with different concentrations of CBD. The same sample was loaded on VAMS^®^ tips and the remaining was centrifuged in order to obtain plasma with same drug concentration. Both were then analyzed by mean of the same LC–MS/MS method. One limitation of the study was that only one clinical pharmacokinetic curve (obtained from one volunteer), was reported.

It is of interest to note that the analytical methods presented in the herein reported studies show a number of similarities to one another, for example all studies used LC–MS/MS and all with basic mobile phases. This demonstrates easy and feasible use of TDM for ASMs from extracts of VAMS^®^ tips. Indeed LC-MS/MS instrumentation may be necessary for measuring extracts from VAMS^®^ tips because of the improved sensitivity and specificity of this technique compared to other platforms such as immunoassay. Moreover, the organic nature of solvents used for the extraction of samples does not allow the use of immunometric or colorimetric techniques without removal of the organic solvent. To adapt the use of this particular micro method to these techniques it would be necessary to develop other extraction methodologies.

### 2.3. Stability

The effect of the stability of ASMs on VAMS^®^ samples have been investigated by a number of groups across a range of temperatures (−60 °C, 37 °C, room temperature, 4 °C, –20 °C and −78 °C), and durations (1–60 days). The results indicated that time and temperature have different effects on specific ASMs and are summarized in Supplemental Material Table S1.

Velghe and al. [29] reported that VPA, PB, PHT, CBZ, and CBZ-E, stored at temperatures ranging from 4 °C to 60 °C for four days or one week, showed a percentage difference from fresh sample did not exceed ±15% except for the CBZ metabolite (CBZ-E). Indeed, when treated at 4 °C or −20 °C after 4 days and at 60 °C after one week the stability bias for the metabolite exceeded ±15%. Moreover, after one month, there was a worsening of stability for VPA at all temperatures. Changes in the stability of PB and CBZ-E were also reported at 60 °C. In another study, Velghe and colleagues [26] went one-step further, testing clinical samples collected in Uganda that were mailed to laboratories in Belgium and finally stored −20 °C. To assess stability, QC samples, stored at −20 °C, in zip-closure plastic bags containing desiccant gel, were examined after storage times ranging from 4 days to 31 days. QC samples with nine leftover hospital patient samples were also examined after 93 and 186 further days of storage. Concentration changes were within acceptable limits (±15%).

Canisius et al. [28] concluded that their stability study enabled an accurate detection of a wide variety of ASMs from VAMS^®^ extracts within 2 days post sampling. However, they also reported that ETS, LTG, OXC, PB, PHT, PGB, and PRM appeared to suffer negative biases as a result of a lack of temperature control, showing a loss of more than 15% in concentration when exposed at room temperature for 1 days, 2 days, 3 days, and 7 days. In particular, OXC seems completely degraded after 7 days. There was instead an improvement of stability when the VAMS^®^ samples were stored at –20 °C for 1days and 7 days: a decrease of degradation was observed for LTG and PHT but still less than –15% of percentage difference from day 1. This demonstrates a clear improvement of stability on dried VAMS^®^ compared to liquid blood, observed when samples were stored at 37 °C for 2 h and 48 h where all differences from day 1 are within ±15%. These data were partially in accordance with D’Urso et al. [30] where all compounds tested on VAMS^®^, stored at –20 °C, 4 °C, 10 °C, and 37 °C for 10 days, were within ±15%.

Stability studies have thus suggested that ASMs on VAMS^®^ were stable until one week at controlled temperature, allowing the potential implementation of VAMS^®^ assays from remotely collected samples. However, it is necessary to carry out a further stability investigation for some specific compounds, such as OXC focusing on the effect of the physico-chemical properties of both the drug and microsampler surface.

Moorthy et al. [23] tested VAMS^®^ tips under very different time/temperature combinations (summarized in Table S1). The authors found an acceptable stability for CBD at temperatures below 0 °C, in the autosampler at 10 °C for 24 h and for QC samples stored for 1 week sealed in a dedicated environment. An acceptable stability was also demonstrated also for post-extracted samples when stored at −80 °C.

Pigliasco et al. [24] also studied the stability of CBD on Mitra^®^ devices at 1 weeks and 4 weeks at two different temperatures (−20 °C and 25 °C) with satisfactory results at a range of concentrations. In fact, the reported variation of accuracy and CV varied between 90–99% and between 3–8% respectively.

The stability studies have shown that Mitra^®^ devices with VAMS^®^ tips allow the exchange and storage of ASMs samples between the patient and the laboratory, even over long distances and/or over longer periods, within acceptable stability limits. For this reason, the aforementioned stability studies were of the utmost importance, to demonstrate future implementation of robust assays from remotely collected samples.

Data collected in the studies reviewed in this paper, clearly demonstrate that Mitra^®^ devices with VAMS^®^ tips meet this need in both experimental and real-life settings. The stability of OXC remains a matter of debate and further studies will certainly be necessary, possibly on large numbers of real finger pricking samples. However, OXC, although pharmacologically active, is the pro-drug of 10-OHOXC, as it is rapidly metabolized to the active metabolite of OXC. Usually, OXC concentration is very low or not detectable in patients’ plasma. Therefore, most laboratories do not measure OXC routinely.

### 2.4. Hematocrit Effect

One of the main advantages of VAMS^®^ is that accurate volumes of blood can be collected independent of HCT values. To measure the HCT effect, a comparison of peak areas of compound(s) spiked into blood samples with a range of HCTs values (typically 25–65%) has been performed. The percentage recovery of these samples is compared to corresponding blank blood samples extracted under the same conditions with standards spiked in post extraction.

D’Urso et al. [30] observed absence of a significant HCT bias across all drugs and HCT levels (35%, 45%, and 55%) tested. Similar results were collected by Velghe and co-workers [29], that investigated a wider range of HCT values (21%, 42%, 52%, and 65%). It was observed that even when high HCT values seem to correspond to a lower recovery, there are not statistically significant differences between the different HCTs, except for the recovery of VPA at 62% HCT compared to 42% in samples prepared by pipetting 10 µL onto the samplers. In the more recent study, Velghe et al. [26] excluded HCT effect on the observed median HCT level of 38.1% (range 20.9–47.9%) of their patients (data shown in Supplemental Digital Content 1, http://links.lww.com/TDM/A381 (accessed on 10 February 2021).

Canisius et al. [28] evaluated HCT effect by calculating the percentage of deviation recovery against HCT values: HCT value varied from 30% to 55% and more than 90% of measurements were within 15% of deviation (OXC was excluded). Finally, the HCT effect was not investigated by Pigliasco, F et al. [24] assuming from previous studies that VAMS^®^ TDM is not influenced by this bias.

Moorthy et al. [23] evaluated the impact of three HCT levels (20.5%, 39.9%, and 67.2%) on the quantitation of CBD. CBD had a matrix effect of 100–108%, across two concentration levels thus demonstrating that CBD had optimal recovery and minimal matrix effect following extraction from Mitra^®^ devices with VAMS^®^ tips employing the described method.

As discussed earlier, Mitra^®^ devices with VAMS^®^ tips have been developed to solve spot area mediated HCT biases seen from DBS post punch extractions. Therefore, the exclusion of a HCT effect represents a crucial point of these method validations because if the contribution of the HCT is considered null or limited, a validation of robust assays across a range of HCTs is possible. Indeed, all studies reviewed within, clearly show HCT independent assays on Mitra^®^ devices with VAMS^®^ tips for measurement of ASM concentrations in whole blood over a wide HCT range is possible.

### 2.5. Blood-to-Plasma Ratio

VAMS^®^ concentrations were compared with serum/plasma concentrations. Velghe and Stove [29] revealed that blood-to-plasma ratio (R) of VPA, PB, PHT, and CBZ were in line with published data, despite the small number of measures obtained; D’Urso et al. [30] confirmed the results for PB and CBZ but obtained a different result for PHT (R = 1.1 ± 0.2), similar with Tamura et al. [33]. D’Urso et al. also measured blood-to-plasma ratio for several antiseizure medications separating those with ratio around 1 from those with ratio different than 1 as evidence of the different capability of binding to red blood cells (RBC). As an example, in this study authors found R = 2.7 ± 0.8 for ZNS reflecting the fact that ZNS is well known to bind substantially to RBC [34,35]. R > 1 was documented also for TPM, LTG, and CBZ-E with R-values similar at those reported previously [36,37,38,39]. It is already known from a study published from Patsalos P.N. [40] on PMP PK using radiolabeled drug that PMP has a blood/plasma ratio covering a range from 0.55 to 0.59, as expected for drugs with a very high plasma protein binding (>95%). In their study D’Urso et al. [30] found R = 0.6 ± 0.1 for PMP, very similar to the reported values.

As discussed in Section 2.1 Velghe et al. [26] had published an article as application of previously validated method, measuring the levels of VPA, PB, and CBZ in patients with Nodding syndrome, from Uganda and Democratic Republic of the Congo, comparing also VAMS^®^ with dried blood spots (DBS). This study confirmed the blood-to-plasma ratios already published but with some differences between the method for VAMS^®^ measure and DBS and serum concentration. Authors also advice that the analytical method must be taken in account (immunoassay vs. LC–MS/MS). Velghe and colleagues, comparing DBS and VAMS^®^ samples, reported a lower variability in incurred sample reanalysis test for DBS. The authors found an overestimation of VAMS^®^ concentrations compared with DBS concentrations that most relevant especially for samples containing PB.

Canisius et al. [28] did not investigate blood-to-plasma ratio.

Regarding CBD, only Moorthy and colleagues [23] reported data on this issue. These authors studied CBD partitioning in both extracted plasma and Mitra^®^ devices with VAMS^®^. R-values measured in plasma ranged from 0.714 to 0.775, showing a good correlation with that found measuring R on Mitra^®^ devices with VAMS^®^ as the reported R for VAMS^®^ ranged from 0.696 to 0.827.

Data on blood/plasma ratio are discussed in more detail in Section 3, Discussion.

## 3. Discussion

The new microsampling technique named Mitra^®^ devices with VAMS^®^ technology, characterized by the ability of collecting fixed-volume capillary blood has been extensively tested for ASMs TDM with good results, for most of the drugs tested.

In the six studies included in this review only three [24,29,30] present data on a full method validation according European Medicine Agency (EMA) and Food and Drug Administration (FDA) guidelines [31,32], the other representing an application of conventional method for plasma or serum. All presented analytical methods are within limits acceptability.

Stability tests were performed from all authors, obtaining useful considerations for real-life shipping and storage. Of interest is the study of Velghe and colleagues [26] in which storage and transport of real samples of finger pricking samples, between very distant places, were simultaneously examined.

HCT effect was investigated, mainly by Velghe [29], D’Urso et al. [30], and by Moorthy et al. [23]. Canisius et al. [28] studied the impact of HCT value as the deviation from recovery extraction; on the other hand, it represents the application with the largest number of patients and therefore provides useful information about VAMS^®^ usage.

The results of blood-to-plasma ratio offer important consideration on the reference values in blood, if available, and on the correct construction of the calibration curves. This critical issue has been investigated in a recent paper by Vincze et al. [41]. Authors performed multiplex analysis (of 14 different drugs) starting from VAMS^®^ devices using calibrators and controls, either in liquid or dried serum or whole blood, with good performances. For those laboratories that are planning to use VAMS^®^ technology, this study is important because it clearly shows that dried serum calibrators allow existing TDM methods, avoiding the need of preparing homemade whole blood calibrators and controls, and hence reducing laboratory burden with higher accuracy and precision.

It is our opinion that VAMS^®^ can be an excellent tool to study the distribution of a drug between plasma and red blood cells, providing an additional tool for pharmacokinetics and TDM studies especially in clinical or in trial settings. A clear demonstration of this fact arises from studies included in the present review and from previous studies, as the one published by Kita and Mano in 2017 [42] on tacrolimus RBC/plasma drug partitioning. However, we must emphasize that before these particular devices can be used for this kind of study, a much larger amount of data will need to be collected to allow for a more accurate determination of whole blood/plasma ratios for the different ASMs. Moreover, for those ASMs where the blood/plasma ratio deviates from 1, a correction factor is needed to calculate a plasma concentration equivalent. We suggest that each laboratory with extensive experience in the TDM of ASMs should derive R-values, obtained from their in-house analytical method, on large numbers of samples, so that a consensus on conversion factors can be reached in the future. However, this approach does not preclude that for ASMs with highly variable R, such as TPM and ZNS, it may be difficult to achieve this goal.

Only two methods refer to capillary blood collected from finger pricks, while the others are based on venous blood collected by dipping into EDTA tube; this could cause a bias due to intrinsic differences between capillary and systemic blood. Moreover, the differences between analytical methods based on plasma/serum samples or whole blood should be taken into account, especially when immunometric methods are applied for the comparison (Table 3).

One other observation that has been seen with blood-to-plasma partitioning is that it can be concentration- and time-dependent. Bailey and co-workers [43] published a model in 2020 which describes the influence of blood-to-plasma ratio and percent HCT on analyte concentrations in whole blood. In doing so, they derived an equation to try to scale more accurately data from whole blood compared to plasma. When they tested their equation, they observed that post scaling, the differences in PK endpoints for a specific analyte tested were much reduced. This then allowed them to demonstrate a PK bridge with the analyte they tested between the matrices they investigated (including plasma and dried blood). In addition, concentration and time from intake effect on TPM blood-to-plasma partitioning was earlier highlighted by Shank et coll. [36] and further discussed in the work of D’Urso et al. [30].

These devices must be considered also with regard to their cost and costs related to their use, including costs for staff training and transport/storage. Today costs may be higher to that related to conventional venipuncture, thus reflecting the fact that Mitra^®^ devices with VAMS^®^, so fare, are not widespread in clinical practice and, their production is still low, compared to devices needed for other sampling techniques.

During 2019 the International Association for Therapeutic Drug Monitoring and Clinical Toxicology (IATDMCT) [44] published an official guideline to give clear indications on how use dried blood spot (DBS) and other microsampling devices (including volumetric absorptive microsampling devices) for TDM and for purposes other than TDM. This guideline defines the parameters necessary for the validation of quantitative DBS-based methods.

The recommendations contained in this guideline arise from the critical analysis of already published indications such those on bioanalytical method validation guidelines issued by EMA and FDA [31,32] the guideline for measurement procedure comparison provided by the Clinical and Laboratory Standards Institute (CLSI) [45] and other papers focused on methods specifically developed for analysis based on dried matrix [46,47,48,49]. The principal focus of this guideline is to harmonize dried sampling techniques and subsequent chromatographic techniques for TDM purposes. A section of the same guideline is dedicated also to the analysis of samples obtained through volumetric absorptive microsampling based on VAMS^®^ technology. The application of microsampling for purpose other than TDM is also investigated.

The validation section compares the analytical validation and the clinical validation assessing the need of demonstrating equivalence between DBS-based (or other microsampling devices based on dried blood) results and results obtained in the classical matrix. In addition, guidance is given on the application of validated methods in a routine context.

A final consideration that must keep in mind is that sampling with Mitra^®^ devices with VAMS^®^ may be performed at home, at the patient’s convenience, and then may be sent by mail. This may represent a further step toward healthcare democratization, as described by Neumaier and Watson [50], and towards the transformation of the concept of “therapeutic drug monitoring” into the more appropriate “*therapeutic drug management*”, aimed at an ever-greater possibility of personalized medicine.

## 4. Conclusions

Volumetric Absorptive Microsampling using Mitra^®^ devices based on VAMS^®^ technology is bringing a revolution in the sampling, pre-treatment, and analysis of biological fluids, especially regarding pharmacokinetic and toxicological studies.

In addition, VAMS^®^ has proved less invasive and therefore is helping in therapeutic drugs monitoring by increasing subject recruitment and retention.

Despite all the advantages already demonstrated, other points (analyte stability, repeated analysis, correlation between plasma and blood concentrations) need to be clarified before the technique will be accepted as a routine bioanalytical procedure. This approach could be adopted as the sampling method of choice in several areas, not only for drug monitoring, but also for toxicokinetic and clinical studies, as well as other fields such as forensics purpose. Further studies are needed to validate this sampling method on real patient finger/heel pricking and to assess the clinical usefulness of the new sampling method.

## Figures and Tables

**Table 2:** Summary of chromatographic conditions, ionization sources, and validation guidelines applied for LC–MS/MS method validation.

Analytes	Mobile Phase	Ion Source	Flow Rate (mL/min)	Eluition Mode *	Column	Run Time	Validation Guidelines	Ref
5 ASM ^1^	(A) 5 mM ammonium acetate;	ESI	1.4	Gradient (%B 20–98)	Chromolith^®^ RP-18 endcapped (100 mm × 4.6 mm, particle size nd)	4 min ESI- method	EMA [31]	[26]
	(B) 5 mM ammonium acetate in acetonitrile/water (95/5, *v*/*v*)					6 min ESI+ method	FDA [32]	[29]
16 ASM ^2^	(A) 0.1% formic acid;	H-ESI	0.25–0.5	Gradient (%B 0–98)	C18 Hypersil Gold (50 mm × 2.1 mm, 1.9 μm particle size)	10.3 min	EMA [31]	[30]
	(B) acetonitrile/0.1% formic acid						FDA [32]	
16 ASM ^3^	(A) 10 µmol ammonium acetate/0.1 mM formic acid;	ESI	0.4	Gradient (%B 40–95)	Syncronis C18 (50 mm × 2.1 mm; 1.7 μm particle size)	12 min	In house validation	[28]
	(B) 100% methanol							
CBD	(A) 5 mM ammonium formate/0.05% formic acid;	ESI	0.4	Gradient (%B 50–98)	Waters Acquity UPLC HSS C18 (100 mm × 2.1 mm; 1.8 μm particle size)	6 min	In house validation	[23]
	(B) 100% acetonitrile							
CBD	(A) 0.1% formic acid;	APCI	0.4	Gradient (%B 50–100)	Waters Acquity HSS T3 (150 mm× 2.1 mm; 1.8 µm particle size)	7.5 min	EMA [31]	[24]
	(B) acetonitrile/0.1% formic acid						FDA [32]	

Legend: ^1^ CBZ, CBZ-E, PHT, PB, and VPA; ^2^ 10-OHOXC, CBZ, CBZ-E, ETS, FBM, LCM, LEV, LTG, OXC, PB, PHT, PMP, PRM, RFN, TPM and ZNS; ^3^ 10-OHOXC, BRV, CBZ, CBZ-E, ETS, GBP, LCM, LEV, LTG, OXC, PB, PGB, PHT, PRM, TPM and VPA. ESI: Electrospray ionization; H-ESI: Heated-electrospray ionization; APCI: Atmospheric pressure chemical ionization; nd: Information not available; * Elution gradients are expressed as the initial and final percentage of mobile phase B.

**Table 3:** Cons and pros for the VAMS^®^ procedure.

Drugs	Cons	Pro	Author
**5 ASMs ^1^**	Leftovers samples Few samples for B/P ratio Correlation with immunoassay	Fully validated HCT effect investigated	[29]
**16 ASMs ^2^**	Leftover samples Venous blood	Number of samples Fully validated B/P ratio investigated HCT effect investigated	[30]
**16 ASMs ^3^**	Blood and VAMS^®^ methods not fully validated HCT indirectly evaluated B/P ratio not defined	Number of samples	[28]
**3 ASMs ^4^**	B/P ratio calculated with immunoassay HCT not evaluated Difficult Storage	Finger pricking Number of patients Capillary blood	[26]
**CBD**	Number of patients HCT not evaluated B/P ratio not defined	Fully validated Finger pricking Capillary blood	[24]
**CBD**	Spiked or leftover samples	Fully validated HCT effect investigated B/P ratio investigated	[23]

Legend: ^1^ CBZ, CBZ-E, PHT, PB and VPA; ^2^ 10-OHOXC, CBZ, CBZ-E, ETS, FBM, LCM, LEV, LTG, OXC, PB, PHT, PMP, PRM, RFN, TPM and ZNS; ^3^ 10-OHOXC, BRV, CBZ, CBZ-E, ETS, GBP, LCM, LEV, LTG, OXC, PB, PGB, PHT, PRM, TPM and VPA; ^4^ CBZ, PB and VPA.

## Data Availability

Data sharing not applicable.

## Supplementary Materials

The following are available online at https://www.mdpi.com/article/10.3390/ph14070627/s1, Table S1: summary of stability studies.
