# Supplementary material for: Therapeutic Drug Monitoring of Antiseizure Medications Using Volumetric Absorptive Microsampling: Where Are We?
Source: Pharmaceuticals (Basel). 2021 Jun 29;14(7):627. doi: 10.3390/ph14070627 (PMC8308594; doi:10.3390/ph14070627)
Supplement: Supplementary file 1 [file pharmaceuticals-14-00627-s001.zip › pharmaceuticals-1211652-supplementary.pdf]

Therapeutic drug monitoring of antiseizure medications using volumetric absorptive microsampling: where are we?

Annachiara D’Urso, Marcello Locatelli, Angela Tartaglia, Linda Molteni, Cristian D’Ovidio, Fabio Savini, James Rudge and Ugo de Grazia.

Supplemental material

TABLE S1 Summary of stability studies

Table S1a: Summary of short time stability studies

Analyte	T (°C)	Percentage difference from day 1 (%)				
		D'Urso, A. et al, 2019 [30]		Canisius T.P.I.J.M. et al, 2020 [28]		
		%QC low	%QC high			
		10 d		2 d	3 d	1 w
10-OH-oxcarbazepine (10-OHOXC)	**-20°**	-7.6	-5.1			<±15
	**+4°**	-9.5	-5.2			
	**+37°**	-12.6	-8.6	<±15		
	**+10°**	-4.1	2.3			
	**RT**			<±15	<±15	**>±15**
Brivaracetam (BRV)	**-20°**			**>±15**		
	**+37°**					<±15
	**RT**			<±15	<±15	**>±15**
Ethosuximide (ETS)	**-20°**	-8.7	-8.4			
	**+4°**	-11.7	-10.0			<±15
	**+37°**	-14.5	-7.3	<±15		
	**+10°**	-5.0	-3.3			
	**RT**			<±15	**>±15**	**>±15**
Felbamate (FBM)	**-20°**	0.6	1.2			
	**+4°**	4.4	-2.2			
	**+37°**	-1.7	-0.6			
	**+10°**	-3.4	-11.4			
Gabapentin (GBP)	**-20°**					<±15
	**+37°**			<±15		
	**RT**			<±15	<±15	<±15
Lacosamide (LCS)	**-20°**	-10.2	-3.9			<±15
	**+4°**	-1.9	-8.5			
	**+37°**	-8.6	-4.2	<±15		
	**+10°**	-2.0	-11.4			
	**RT**			<±15	<±15	<±15
Levetiracetam (LEV)	**-20°**	-2.9	-3.1			<±15
	**+4°**	-3.6	-4.0			
	**+37°**	-6.2	-0.7	<±15		
	**+10°**	-6.0	-9.0			
	**RT**			<±15	<±15	<±15
Lamotrigine (LTG)	**-20°**	-9.5	-3.0			<±15
	**+4°**	-5.9	-4.4			
	**+37°**	-3.2	-3.3	<±15		
	**+10°**	2.2	-9.5			
	**RT**			<±15	**>±15**	**>±15**
Oxcarbazepine (OXC)	**-20°**	7.3	-2.7			<±15
	**+4°**	-13.1	3.3			
	**+37°**	-1.4	-14.7	<±15		
	**+10°**	-3.5	-8.4			
	**RT**			**>±15**	**>±15**	**>±15**
Perampanel (PMP)	**-20°**	-3.5	-3.5			
	**+4°**	3.2	3.2			
	**+37°**	-11.2	-11.2			
	**+10°**	-3.3	-0.5			

Pregabalin (PGB)	**-20°**					<±15
	**+37°**			<±15		
	**RT**			<±15	<±15	**>±15**
Primidone (PRM)	**-20°**	-0.4	-9.4			<±15
	**+4°**	2.5	-8.0			
	**+37°**	-2.4	-4.8	<±15		
	**+10°**	-3.0	-3.4			
	**RT**			<±15	<±15	**>±15**
Rufinamide (RFN)	**-20°**	-14.8	-9.4			
	**+4°**	-12.1	-11.3			
	**+37°**	-12.9	-6.8			
	**+10°**	-3.2	-2.6			
Topiramate (TPM)	**-20°**	-8.2	-13.8			<±15
	**+4°**	3.0	-9.9			
	**+37°**	-9.0	-12.4	<±15		
	**+10°**	2.6	-6.4			
	**RT**			<±15	<±15	<±15
Zonisamide (ZNS)	**-20°**	-6.1	-12.2			
	**+4°**	-1.4	-13.1			
	**+37°**	-1.2	-14.1			
	**+10°**	0.9	-4.4			

Legend: d) days; **w)** weeks; **QC low)** low level Quality Control; **QC high)** high level Quality Control. **Red bold numbers** represent numbers exceeding allowable stability limit (<±15). Data are expressed as mean percentage difference in concentration with respect to day 1.

Table S1b: Summary of long time stability studies

Analyte	T (°C)	Percentage difference from day 1 (%)														
		Velghe, S., Stove C.P., 2018 [29]						D'Urso, A. et al, 2019 [30]		Canisius T.P.I.J.M. et al, 2020 [28]			Velghe, S. et al, 2020 [26]			
		4 d		1 w		1 mo		10 d		2 d	3 d	1 w	3 mo		6 mo	
		%QC low	%QC high	%QC low	%QC high	%QC low	%QC high	%QC low	%QC high				%QC low	%QC high	%QC low	%QC high
Carbamazepine (CBZ)	-20°	-0.4	-3.6	-13.4	-14.9	-1.8	1.7	-3.8	-9.3			<±15	5.1	-1.5	-5.5	-4.9
	+4°	-0.3	4.7	-13.9	-13.5	-3.6	-0.9	-5.2	6.6							
	+10°							-0.7	1.8							
	RT	-9.2	-5.5	-13.7	-8.8	-4.6	3.2			<±15	<±15	<±15				
	+37°							-6.6	5.1	<±15						
	+60°	-12.1	-5.1	-10.9	-12.3	-10.8	0.9									
Carbamazepine -epoxide (CBZ-E)	-20°	17.5	0.0	5.0	-0.8	5.1	-0.2	-9.9	-7.6			>±15				
	+4°	15.9	5.3	6.9	-5.4	11.4	-1.7	-1.9	-9.5							
	+10°							-1.1	-4.1							
	RT	7.5	-4.3	5.9	-0.1	4.0	-4.1			<±15	<±15	<±15				
	+37°							-9.6	-12.6	<±15						
	+60	2.9	-9.2	-5.9	-15.3	-23.6	-26.1									
Phenobarbital (PB)	-20°	8.3	-6.2	-3.1	-11.8	13.2	7.4	-7.4	-8.6			<±15	12.5	4.1	-6.9	-10.4
	+4°	5.2	-0.4	-7.2	-13.6	7.0	5.9	4.3	-2.0							
	+10°							2.5	-3.3							
	RT	-5.3	-6.2	-6.0	-10.5	5.1	0.3			<±15	>±15	>±15				
	+37°							4.8	-2.4	<±15						
	+60°	0.0	-7.8	0.6	-13.0	15.5	6.8									
Phenytoin (PHT)	-20°	1.5	10.0	-6.1	5.2	9.5	7.4	4.3	3.1			>±15				
	+4°	-0.8	12.4	-8.4	3.8	11.8	7.8	-2.0	-0.4							
	+10°							-1.8	-2.8							
	RT	-8.4	4.2	-8.3	9.6	11.9	9.2			<±15	>±15	>±15				
	+37°							-0.3	-4.9	<±15						
	+60°	-4.9	3.0	-10.3	2.3	11.0	13.3									
Valproic acid	-20°	12.1	0.3	11.8	-4.4	15.5	17.6					<±15	15.2	10.3	-1.4	-1.4

(VPA)	**+4°**	2.1	2.0	11.8	-8.0	**15.1**	**18.0**									
	**RT**	-6.0	-9.3	11.5	0.7	**17.6**	10.4			<±15	<±15	<±15				
	**+37°**									<±15						
	**+60°**	2.3	-2.3	6.3	-8.0	**16.3**	**28.1**									

Legend: d) days; **w)** weeks; **m)** months; **QC low)** low level Quality Control; **QC high)** high level Quality Control**. Red bold numbers** represent numbers exceeding allowable stability limit (<±15). Data are expressed as mean percentage difference in concentration with respect to day 1.

Table S1c: Summary of CBD stability studies

**Analyte**	**T (°C)**	**Percentage difference from day 1 (%)**															
		**Moorthy, G.S. et al, 2019 [23]**								**Pigliasco, F. et al, 2020 [24]**							
		**1 w**		**2 w**		**1 mo**		**2 mo**		**1 w**		**2 w**		**3 w**		**4 w**	
		**QC low**	**QC high**	**QC low**	**QC high**	**QC low**	**QC high**	**QC low**	**QC high**	**LLOQ**	**QC high**	**LLOQ**	**QC high**	**LLOQ**	**QC high**	**LLOQ**	**QC high**
Cannabidiol (CBD)	**-78°**			94.6 ± 9.93 (envelope)	92.6 ± 4.64 (envelope)			106 ± 3.04 (autorack)	109 ± 4.18 (autorack)								
	**-20°**			92.4 ± 5.36 (envelope)	100 ± 10.2 (envelope)	101 ± 5.08 (autorack)	113 ± 0.658 (autorack)			91% (6%)	92% (7%)	99% (8%)	97% (6%)	98% (4%)	99% (3%)	99% (3%)	98% (8%)
	**+4°**			90.2 ± 11.4 (envelope)	101 ± 8.13 (envelope)	**80.1 ± 22.3 (autorack)**	86.9 ± 3.66 (autorack)										
	**RT**			105 ± 8.07 (envelope)	89.2 ± 7.18 (envelope)	112 ± 6.13 (autorack)	**116 ± 5.12 (autorack)**			97% (8%)	94% (1%)	94% (6%)	91% (4%)	98% (4%)	90% (3%)	97% (6%)	91% (8%)
	**+40°**	112 ± 5.42 (envelope) 111 ± 5.85 (autorack)	107 ± 5.92 (envelope) 105 ± 5.63 (autorack)														

Legend: w) weeks; **m)** months; **LLOQ)** Quality control set at lower level of quantification; **QC low)** low level Quality Control; **QC high)** high level Quality Control**.** **Red bold numbers** represent numbers exceeding allowable stability limit (<±15). Data are expressed as: Accuracy±CV(%) in ref [23] and accuracy and CV percentage in ref [24]. Stability data in [23] were obtained either putting VAMS in a transport bag (envelope) or in a VAMS dedicated storage rack (autorack).
